# Extracellular Traps: An Ancient Weapon of Multiple Kingdoms

**DOI:** 10.3390/biology9020034

**Published:** 2020-02-18

**Authors:** Ariane Neumann, Graham Brogden, Maren von Köckritz-Blickwede

**Affiliations:** 1 Department of Clinical Sciences, Division of Infection Medicine, Lund University, Baravägen 27, 22184 Lund, Sweden; ariane.neumann@med.lu.se; 2 Department of Physiological Chemistry, University of Veterinary Medicine Hannover, Buenteweg 17, 30559 Hannover, Germany; Graham.brogden@twincore.de; 3 Research Center for Emerging Infections and Zoonoses (RIZ), University of Veterinary Medicine Hannover, Bünteweg 17, 30559 Hannover, Germany

**Keywords:** neutrophil extracellular traps (NETs), defence mechanism, tree of life, innate immunity, conserved mechanisms

## Abstract

The discovery, in 2004, of extracellular traps released by neutrophils has extended our understanding of the mode of action of various innate immune cells. This fascinating discovery demonstrated the extracellular trapping and killing of various pathogens by neutrophils. During the last decade, evidence has accumulated showing that extracellular traps play a crucial role in the defence mechanisms of various cell types present in vertebrates, invertebrates, and plants. The aim of this review is to summarise the relevant literature on the evolutionary history of extracellular traps used as a weapon in various kingdoms of life.

## 1. Introduction

**Figure 1:**
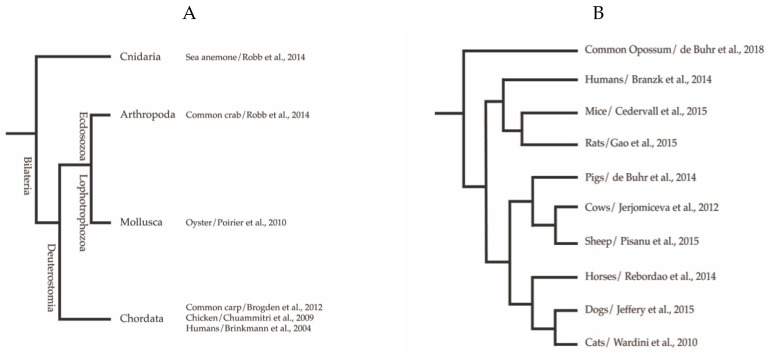
Phylogenetic distribution of extracellular traps among the (**A**) Animalia kingdom, with emphasis on Mammalia (**B**) with example references. Phylogeny was adapted from Kato et al. [21]. Branch length not calibrated, and root position not determined.

After its discovery in 2004 [1], the formation of extracellular traps (ETs) by neutrophils had mainly been studied in vertebrates with a focus on human and mice, however, subsequent research revealed the neutrophils from different mammalian species form ETs, for example, horses, dogs, sheep, mice, as well as from invertebrates [2,3,4,5,6]. ETs are characterised as released DNA associated with histones and granule proteins, which form an extracellular web-like structure (Figure 1). These structures are able to function as an immune defence mechanism through entrapment and killing of certain microbes [7]. In addition to neutrophils, ETs are formed by other immune cells such as mast cells, monocytes, macrophages, and eosinophils when stimulated with mitogens, cytokines, pathogens, or interaction with neighbouring cells, for example, platelets [8]. On the one hand, ETs exhibit a protective role against several invading microbial pathogens as an antimicrobial defence strategy; but on the other hand, an excessive ET release can lead to detrimental effects, for example, thrombosis [9], tissue damage [10], autoimmune diseases [11], and cancer [12], a phenomenon with high clinical relevance [13]. Finally, the fine balance between protective ET formation, and subsequent efficient elimination by the host defines the protective versus the detrimental consequences of ET formation during various diseases. A better understanding of the processes of ET formation by different cell types and different hosts is still needed to target ETs for therapeutic interventions. 

On the basis of findings from several studies mostly performed with human and murine neutrophils, the following three different pathways that lead to the formation of ETs by innate immune cells have been identified: (1) Release of nuclear DNA by ETosis, a suicidal cell death associated with the rupture of the nuclear membrane prior to cell lysis [14,15]; (2) vesicular release of nuclear DNA by viable cells [16,17]; and (3) release of mitochondrial DNA [18,19]. However, the exact molecular mechanisms leading to one or the other phenotype of ET formation has still not been entirely clarified. A group of renowned scientists and experts on NETs has recently published an opinionated review on the subject due to the abundance of available data that has also led to some confusion in the NET/ET research community because of contradictory results and divergent scientific concepts, for example, the molecular pathways of ET formation or the origin of the DNA that forms the ET scaffold [20]. There is a strong consensus about the composition of ETs among the findings that NETs contain a high amount of granule proteins, for example, cell-type-specific proteases and other antibacterial molecules that are associated with DNA-histone complexes. However, it is still unclear how different triggers or pathways have led to phenotypical differences about the source of DNA or viability of the ET-releasing cell.

Comparison of ET phenotypical differences between host species in relation to evolutionary aspects, especially by comparing data from phylogenetic groups, would help to understand the pathways of ET formation in more detail.

## 2. Extracellular Trap Formation Is a Conserved Mechanism

**Figure 2:**
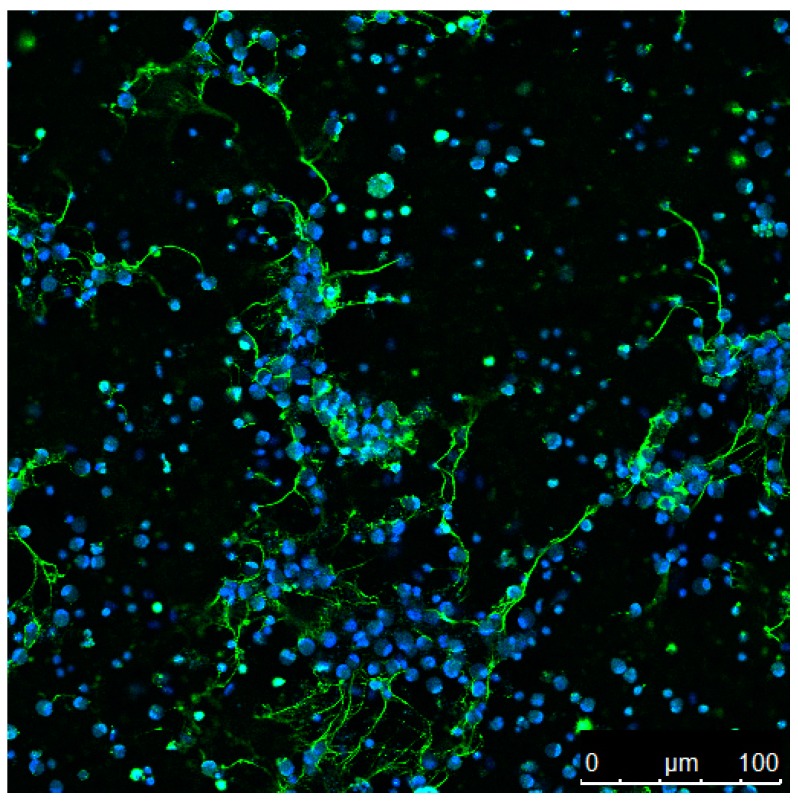
Carp kidney derived neutrophils. Nuclei are stained with DAPI (blue) and extracellular traps with an antibody against H2A-H2B-DNA complexes (green).

The formation of ETs has been reported for several vertebrate species including mammalian, avian, and fish species. Recent publications continue to add to the extensive number of invertebrates, as well as plant species possessing cells capable of ET formation. Figure 1 provides an overview of the kingdoms in which this host defence mechanism has mainly been described so far. Although ET release is observed in response to a variety of biological and chemical stimuli, as summarised in Table 1, the extrusion of antimicrobial DNA/histone complexes seems to be well conserved throughout the tree of life. An example is shown in the immunofluorescence micrograph (Figure 2) that depicts ETs from fish neutrophils. 

In the following sections, we present a summary about studies performed with ET-releasing immune cells from various species among multiple kingdoms (Figure 2); additionally, we give information about the multitude of ET triggers, hypothesised pathways involved, and host proteins associated with the extracellular DNA structures (Table 1). 

## 3. Mammalian Neutrophil Extracellular Traps

### 3.1. Extracellular Traps in Homininae

Brinkmann and colleagues first described the formation of neutrophil extracellular traps (NETs) in 2004; the focus of the subsequent investigations was the host immune protection carried out by NET fibers. Still, several studies have shown the beneficial effects of NETs against various pathogens such as *E. coli*, *S. aureus*, *P. aeruginosa*, *Aspergillus fumigatus*, *Borrelia burgdoferi*, *Human immunodeficiency virus*, *Myxoma virus*, *C. albicans*, *Entamoeba histolytica*, *GBS*, *GPAC*, and *T. gondii* [22,23,24,25,26,27,28,29,30]. Interestingly, NET formation, instead of phagocytosis-mediated killing, was described to occur depending on the size of the invading pathogen and was named size-selective NETosis [95]. However, several inflammatory molecules have also been described to modulate NET formation and various pathways are known which could depend on the specific inducing stimulus. Kenny and colleagues confirmed these findings by suggesting that neutrophils use different pathways to produce and release NETs, depending on the activator used [29]. 

Initially, the protective effect of NETs was confirmed in patients suffering from chronic granulomatous disease (CGD) [14]. CGD patients are severely immunodeficient, have recurrent infections, often with opportunistic pathogens, and have a poor prognosis. Conforming to this, neutrophils from those patients showed impaired killing of extracellular *S. aureus* and impaired capacity to make NETs. Later, the protective effect of NETs was also confirmed in regard to pathogen-induced sepsis, because neutrophils from septic patients showed impaired NET release [96], especially for non-sepsis survivors [97]. In contrast to non-survivors, neutrophils from sepsis-surviving patients were more capable of releasing NETs and granular proteins [97]. An increase in autophagic vacuoles was correlated to the increased NET release in sepsis survivors, indicating a priming of NET formation by autophagy. Interestingly, the inhibition of NET release by the application of anti-inflammatory drugs, and also NF-κB inhibitors, resulted in increased bacteraemia [98]. 

In addition to the protective antimicrobial effects, aggregated ETs are also able to degrade pro-inflammatory cytokines, and thus have been shown to resolve the inflammation in gout patients [99]. However, NETs have been repeatedly described as a double-edged sword [13,98,100], especially in cases where the host is not able to get rid of the ET fibres in the body. Evaluating the overwhelming response of NETs in sepsis or acute pancreatitis revealed a rather detrimental impact of web-structures on the host organism due to organ damage [10,101]. The attenuation of NETosis by inhibition or knockout of factors involved in the generation of NETs, such as neutrophil elastase (NE) or peptidyl-arginine-deiminase (PAD4) [35,102], drastically reduced organ damage in liver vasculature [103]. Recently, a connection between NET formation and RIPK1/3/MLKL-dependent necroptosis leading to endothelial tissue damage was reported [104]. In addition, mitochondrial damage was described after the exposure of dendritic cells to NETs [105]. It was speculated that NETs could be used as a marker for community-acquired pneumonia, since an increase of NETs present in serum was correlated to clinical instability, prolonged length of hospital stay, and mortality [106]. As multiple tumour types displayed the ability to facilitate the release of NETs from circulating neutrophils, it was hypothesised that, in contrast to the findings by Millrud and colleagues [107], neutrophil responses in the context of tumour progression could be catastrophic to the host [12]. Another study, however, supports these findings, whereby inhibition of NETs by chloroquine decreased hypercoagulability, and thus cancer-associated thrombosis [108]. These are some examples summarising the work describing the role of protective versus detrimental effects of ETs in humans and highlights the need for future studies to better understand the underlying mechanisms in ET formation.

### 3.2. Murinae

Although Hominidae and Murinae are phylogenetically closely related, they have evolved to become very different organisms because they have adapted to different environments [109]. Thus, mice and rats often respond to experimental interventions in ways that differ strikingly from humans and some authors even discuss that mice are invaluable for studying biological processes that have been conserved during the evolution of the rodent and primate lineages. In addition, regarding ET formation in rodents and human, cells have been shown to react differently. As an example, in human peripheral blood neutrophils, pharmacological or endogenous inhibition of MPO decreased NETosis [110,111], whereas mouse-derived neutrophils did not show the same phenotype in regard to the pharmacological inhibition [110]. Thus, these authors mention, that there is a need for caution in extrapolation to humans from studies on murine NETosis [110]. 

However, the investigation of ET formation in regard to pathogens or also pathological conditions, such as autoimmune responses or cancer has been widely explored in mice [9,37]. For example, in addition to earlier findings of murine NET release in response to *L. monocytogenes* or pneumococci [42,43], a study by Bonne-Année et al. (2014) showed that murine bone marrow derived neutrophils released DNA in response to helminth parasites [5]. The interaction of murine neutrophils and *C. albicans*, an opportunistic yeast often found in immuno-compromised individuals, was investigated by Hopke et al. [44]. A rapid NET production on the yeast hyphae was found, which was hypothesized to contribute to beta-glucan unmasking and chitin deposition [44]. 

Similarly, as shown for human NETs [112], murine NETosis is also triggered by platelet interaction [34] via P-selectin [33] and the involvement of PAD4 [35]. NETs were reported in thrombi of mice after inferior vena cava stenosis, in the context of atherosclerosis and a sepsis model [9,34,35]. In contrast to mice, rats are not used as frequently for studies on ET formation, mainly because of the limited availability for genetic manipulation. However, the involvement of NETs in thrombus formation was supported by a study conducted by Boettcher and colleagues, in 2017, in rats, which found elevated levels of extracellular DNA during testicular torsion in male rats [36]. The authors suggested that NETs display procoagulant properties, providing a scaffold for thrombus formation and triggering platelet activation [36]. The testicular cell damage, elevated MPO staining, oxidative stress, and apoptosis were decreased by DNAse I treatment [36]. NETs, often identified by complexes of DNA/histones, were found in ileum mucosa and submucosa of LPS-infected rats and after myocardial ischemia/reperfusion (MI/R) injury [38,39,40]. Administration of DNaseI decreased tissue damage by NETs, reduced infarct size, and reduced the acute inflammation due to LPS induction [38,39,40]. 

It is also important to mention that the key findings related to NETs and their role in autoimmune diseases, for example, systemic lupus erythematosus (SLE) have been made in a rodent model [113,114,115]. Furthermore, it has also been shown that treatment with HMGB1 antibody inhibited the infiltration of neutrophils and, subsequently, NET formation without altering the antibody production in lupus-prone mice [116]. The antibody treatment additionally suppressed the NET manifestation in the glomerulus [116]. The detection of NETs in lesions of patients suffering from MPO-antineutrophil cytoplasmic antibody (ANCA) associated vasculitis (MPO AVV) suggested an additional role of NETs in this autoimmune condition [41]. MPO AVV often occurs in patients suffering from hyperthyroidism, and thus treated with propylthiouracil (PTU) [117]. Injecting rats with NETs triggered by PMA and PTU resulted in MPO-ANCA production, abnormal NET formation, and increased pulmonary haemorrhage [41]. 

Fewer NETs in lesions were also found in mCat mice, a knockout for mitochondrial stress [118], whereby reduced NET formation was associated with a decreased number of MPO-positive cells. The authors described that NET formation was increased in atherosclerosis in aging mice. Suppression of mitochondrial stress decreased NETs in lesions of aging mice, but not in younger mice [118]. Similar to human NETs, a negative impact of NETs in tumour formation was also reported for mice genetically modified for cancer development predisposition [37]. In summary, key insights into the role of ETs during health and disease were made using the rodent model, thus, highlighting their important role in ET research. 

### 3.3. Chinchillidae

In addition to murine models to study several pathological conditions, a chinchilla model has been used to determine the ability of pneumococci to form biofilms in vivo. Live bacteria and pneumococcal communities were found within the meshwork of bacterial and host cell matrix [119]. Although NETs have been described as a host defence mechanism [1], here, the web-like structures seemed to be not involved in bacterial clearance [119,120]. This was confirmed by a recent study that illustrated that the generation of acidic microdomains of *P. aeruginosa*-derived biofilms, and thus resistance to antibacterial treatment, was related to an accumulation of extracellular DNA (eDNA) [121]. 

### 3.4. Caviidae

In the study by Filio-Rodriguez and colleagues, the effect of *M. tuberculosis* was tested on rodent neutrophils using a guinea pig model. *M. tuberculosis* induced NET formation and neutrophil accumulation at the site of infection after 30 min. In the NETs, which entrapped but did not kill *M. tuberculosis,* active MPO and ROS were found to be associated [122]. 

Taken together, these findings indicate a broad spectrum of stimuli triggering the release of NETs from various rodent families, including mice and chinchilla. The context in which NETs are formed includes cancer, autoimmune responses, as well as responses to pathogenic triggers such as diverse bacteria.

### 3.5. Extracellular Traps in Bovidae

#### 3.5.1. Bovinae

Cattle are the most important agricultural species with almost 1.5 billion farmed animals worldwide [123] and infection with pathogens such as *S. aureus* can lead to critical conditions, including clinical mastitis (CM), which has a high economic impact. Initial findings that identified formation of ETs in cattle have been made in the uterus after the addition of spermatozoa in a pathogen-independent manner [124]. Importantly, the increased binding with seminal plasma to neutrophils and NETs reduced the number of free spermatozoa, indicating that sperm transport to the site of fertilization (and thus fertility) was hindered [124].

The exposure of bovine neutrophils to various other bacterial and parasitic pathogens and their components such as *S. aureus*, *M. haemolytica* leukotoxin (*lkt*;), mammary pathogenic *E. coli* (MPEC) P4, *Histophilus (H.) somni*, *E. faecalis*, *S. dysgalactiae* and clinical isolates of *E. coli*, *E. coli* LPS, live sporozoites of *Eimeria bovis*, and sporozoites of *Cryptosproridium (C.) parvum* resulted in the release or enhancement of NET-like fibres or extracellular DNA [45,48,50,51,52,53,54,55]. Several of these pathogens were found entrapped and killed within the NET-like structures, which were formed in a ROS-dependent fashion [47,53,54], whereas others, such as mycoplasma bovis, degrade NETs and utilise a nuclease in order to escape NET-mediated killing [46]. 

Using bovine granulocytes, key discoveries of the interaction of ETs and parasitic infections have been made. *Neospora caninum*, an apicomplexan parasite infecting cattle, induced NETs within 30 min with the parasite becoming entrapped in the structures shortly afterwards [57]. The involvement of several pathways, such as NADPH oxidase, ERK1/2, and p38 MAPK, as well as intracellular Ca^2+^ mobilisation as described for other parasitic stimuli [58], were not seen in this study [57]. Incubation with a CD11b antibody, however, decreased the formation of NETs, indicating a possible interaction of the parasite with the neutrophils. Interestingly, the study reported that a co-incubation of PMNs with the parasites and, subsequent, NET formation, prevented host cell invasion into bovine umbilical vein endothelial cells (BUVECs) [57]. The L3 larvae of *Haemonchus contortus* triggered different types of NETs, with disseminated and aggregated NETs entrapping the parasite larvae [59]. The aggregated NETs also covered most of the larvae, hampering the parasites’ motility [59]. Although *N. caninum* and *H. contortus* viability was not affected by bovine neutrophils, another study showed *T. gondii* were killed by bovine NETs [60]. 

Furthermore, mechanistical studies to identify molecular insights in the pathways involved in bovine ET formation have been performed. Treatment of bovine neutrophils with fluoroquinolone enrofloxacin led to increased protein expression of PAD4 and enhanced levels of citH3 and NET formation [56]. An inhibition of actin and tubulin filaments in bovine neutrophils led to reduced formation of NETs [56], which has also been described for human neutrophils by Neeli and colleagues [125]. Additionally, involvement of NE and MPO enzymatic activity has been described to be involved in bovine NET release [58]. NET release was also shown to be significantly higher in milk as compared with media [52]. Interestingly, d-lactic acid, a metabolite associated with aseptic inflammatory processes, induced NET formation within 30 min of co-incubation [61]. The NETs were positive for citrullinated histone 4 and CD11b, and the mechanism was suggested to be dependent on the MCT1 plasma membrane transporter [61]. D-lactic acid induced DNA/NET release which, then, enabled neutrophil adhesion to endothelial cells via CD11b and ICAM-1. However, further studies need to be performed to compare these ET-phenoytpes with NETosis, vesicular release, or mitochondrial release of ET formation as described for human or murine neutrophils.

Finally, as previously mentioned, a detailed protocol for the isolation of bovine granulocytes to study NETs was published by Baien and co-workers [126]. Notably, the authors show that density gradient centrifugation of K_3_EDTA blood resulted in higher purity of bovine granulocytes as compared with lithium heparin blood. In contrast to water lysis, the NaCl lysis method was recommended by the authors to avoid preactivation of cells, which can occur during hypotonic water lysis. Thus, these data show that the isolation procedure could impact the results significantly. This could also be considered for granulocytes derived from other species.

#### 3.5.2. Caprinae

Other animals belonging to the Bovidae family, such as goats and sheep, have also been reported to release extracellular traps in response to bacterial or parasitic pathogens [4,62,127]. The intracellular protozoan parasite, *Neospora caninum*, induced NET formation in goat neutrophils after 60 min of exposure [127]. The parasites were found entrapped within the NET structures, which were positive for H1, H2A/B, H3, H4, NE, pentraxin, and cathepsin B [127]. The phenotype was blocked by the MPO-inhibitor ABAH but was independent of NADPH-oxidases [127]. In addition, caprine monocytes respond to parasite interaction with the release of extracellular traps, as reported by Perez et al. [63]. NADPH-oxidase-dependent ETosis was described in response to viable sporozoites, sporocysts, and oocysts of *Eimeria ninakohlyakimovae*, with NETs detected with attached parasites after 30 min [63]. Additionally, IL-12 and TNFα were found upregulated [63]. The authors hypothesised that the released DNA structures immobilised rather than killed the parasites. Furthermore, *Eimeria arloingi* triggered the release of ROS-dependent caprine neutrophil ET fibres and were entrapped within the meshwork [62]. Although *E. arloingi* were immobilised within the NETs, this did not affect the viability of the parasites [62]. The induction of caprine NETs by *E. arloingi* was confirmed by Munoz-Caro et al. (2016), who reported colocalisation of extracellular DNA with histones in infected intestinal tissue [49]. Citrullinated histone H3, a typical NET marker for human and mouse NETs [128], was found in close proximity to Eimeria in different stages of replication. Histone citrullination could also be important in the formation of ETs in sheep; these NETs were found in mammary alveoli in response to *S. uberis* infection [4]. *T. gondii*, a parasite found to trigger NETs in human and murine neutrophils [28], was tested for its impact on ovine and bovine neutrophils [60]. The tachyzoites induced NETs in ovine neutrophils after 30 min of co-incubation in a time-dependent manner. Additionally, the parasites were found entrapped within the meshwork [60]. 

### 3.6. Extracellular Traps in Suidae

Infectious diseases in pigs could account for high economic losses, as pork accounts for one-third of worldwide meat production [129]. Bacterial pathogens *P. aeruginosa*, as well as *S. aureus*, also induced NET release in porcine PMNs [65]. The bacteria were found entrapped within the fibre-like structures, which was dismantled by DNase treatment [65]. By co-incubation of pig-derived neutrophils with *S. suis*, an important porcine pathogen, de Buhr et al. (2014) found significant NET formation and entrapment but not killing of the pathogen [66]. Additionally, granule components and DNA/histone complexes were shown to colocalise with DNA in porcine NETs [65,66]. To aid treatment of respiratory diseases in pigs, the administration of immunomodulatory agents such as granulocyte-colony stimulating factor (G-CSF) was investigated [67]. This application did not alter the capacity of porcine neutrophils to release NETs or their MPO activity in response to PMA, ionomycin, or zymosan [67]. Interestingly, it has recently been shown that degraded neutrophil extracellular traps promote the growth of *Actinobacillus pleuropneumoniae (A.pp)* [130], an important porcine pathogen leading to high economic loss [131]. *A.pp,* itself, releases no nuclease, but the authors identified host nucleases as a source that degrades NETs after *A.pp* infection [130]. These data shed light on the detrimental effects of NETs during a host immune response against certain bacterial species that require or efficiently take advantage of degraded DNA material which has been provided by host nuclease or nucleases of other coinfecting bacteria as a source of growth-aiding nutrients.

### 3.7. Extracellular Traps in Equidae

Equine NET formation was analysed with respect to the role of SCGB1 [132], a protein described as being associated with reduced neutrophil migration and decreased ROS production during recurrent airway obstruction [133,134]. Horse neutrophils released NETs in a presumably ROS-independent manner, which were inhibited by the addition of SCGB proteins [132]. Co-incubation of equine PMNs with various pathogens causing endometriosis in mares (*S. equi* subspecies *zooepidermicus*, *E. coli,* and *S. capitis*) resulted in NET release from equine PMNs and all pathogens were found entrapped within the meshwork [2]. The fact that *S. equi* infection resulted in fewer NETs was explained in literature reporting the nuclease-producing evasion of some streptococci [135,136]. Furthermore, sperm motility, as well as fertility, has been reported to be reduced due to the binding of neutrophils to the sperm in the female reproductive tract (FRT) [137]. Interestingly, the incubation of equine neutrophils with sperm induced the formation of NETs and, moreover, resulted in entrapment of the spermatozoa [68]. NETosis was prevented by the addition of seminal plasma (SP) [68], which also reduced the binding between spermatozoa and neutrophils. Low-density neutrophils (LDN), found in the PBMC layer after density centrifugation, displayed a higher rate of Fmlp receptors on their surface as compared with normal density neutrophils [69]. Although their MPO content was the same, LDN were found to be more sensitive to PMA, and thus showed an increased capacity to produce NETs [69]. This finding could be important, since LDN exhibit an increased synthesis of TNFα, IL-6, IL-8 and IFN1 and are found in the blood of horses suffering from lupus and cancer, as well as systemic and local infections [69]. These data correlate well with the finding by Villanueva and colleagues, who associated elevated NET-forming capacity of LDN to lupus [138]. 

Recently, NETs have been found in association with equine recurrent uveitis (ERU) [139]. NETs found in ERU-horses were positive for MPO. More NETs were found in vitreous body fluids and serum samples of horses diagnosed with ERU as compared with healthy horses; and thus, the authors speculated that NETs contribute to the pathogenesis of ERU [139]. Increased NET formation was also detected in airway neutrophils of asthmatic horses [140], which could be diminished by treatment with glucocortisteroid. 

Finally, the apicomplexan parasite, *T. gondii*, has also been shown to trigger NET formation in other mammalian species [28,80], and in donkeys [70]. These NETs were correlated with NE and ROS activity [70].

### 3.8. Extracellular Traps in Carnivores such as Felidae, Canidae, Pinnipedae, and Mustelidae

The incubation of feline neutrophils with the parasitic pathogen *Leishmania* resulted in extrusion of DNA with the parasite being entrapped within the meshwork [73]. It was suggested that an infection with feline leukaemia virus (FeLV) leads to exacerbated neutrophil activation, and thus made these cells less susceptible to other stimuli [73]. Similar to other mammalian neutrophils, cat neutrophils also responded to tachyzoites of *T. gondii* in a ROS-dependent fashion by releasing NETs [71]. Recently, NETs were also found in the endometrium of queens and bitches, suffering from *E. coli*-mediated pyometra [72]. The pathogens were found entrapped in the meshwork, which was positive for NET markers MPO, H2B, and NE [72]. *E. coli* derived LPS induced PAD4-mediated NET formation in canine neutrophils, leading to intracellular citrullination of histone H3 [76], while PMA triggered the release of larger NET areas, with both of these structures stained for DNA, citH3, and MPO [76]. Another study found that well-known NET inducers, namely PMA and PAF, both induced DNA release by canine PMNs and neutrophil elastase was found colocalised with the fibrous structures [3]. Since the study showed elevated levels of circulating nucleosome, a marker for immune-mediated haemolytic anaemia (IMHA), the authors hypothesised a contribution of NETs in the mortality of this disease; although they argued that their results should be viewed with caution due to the low number of patients [3]. The same group recently analysed the involvement of NETs in clot formation [75]. They suggested that NET proteins and DNA contribute to thrombosis in inflammatory diseases. Their study showed that NETs increased the maximum clot formation velocity and delayed lysis. DNAse treatment reduced the effect on the clot lysis, but not on the formation velocity, indicating an involvement of NET associated proteins [75]. It was hypothesised that NET proteins interact with the early components of the coagulation cascade; thus, canine NETs can be used as markers of thrombotic risk [75]. To detect the DNA fibres in canine plasma, the commonly used SytoxGreen DNA-quantification assay [141] was compared with a DNA/histone ELISA [142]. The authors concluded that both assays were suitable, although the ELISA seemed more sensitive [142]. The parasite *N. caninum*, studied for its interaction with caprine and bovine neutrophils, was recently investigated for its impact on NETs derived from the definitive host of the Canus genus [74]. *N. caninum* tachyzoites induced NETs within 30 min of co-incubation, with positive staining for NET markers H3, MPO, and NE used. The NET induction by *N. caninum* was time- and dose-dependent, as well as dependent on MPO, NE, and ROS production [74]. The study showed that ERK1/2, MAPK, and SOCE pathways were involved in the phenotype, and the parasites were entrapped within the NET structures [74]. Recently, two studies showed that canine PMNs also release extracellular traps in response to *Trypanosoma cruzi* [77] and *Dirofilaria immitis* microfilariae, and their L3 stage larvae [78]. Finally, also an overexposure to nickel in the form of nickel nitrate, a commonly used heavy metal material in battery manufacturing, triggered the release of extracellular traps from canine neutrophils [143]. The pathway involved in this phenotype is still unclear [143].

Smaller carnivores such as ferrets (Mustelidae) have frequently been used to study the pandemic potential of emerging influenza viruses [144]. While infecting ferrets experimentally with influenza H1N1 strain, extracellular histone (H3) was found in neutrophil-containing areas in the respiratory tract, which points to a possibility of NET formation in the lumen of infected bronchioles [79]. *Haemophilus (H.) influenzae* is a common otitis media (OM) or middle ear infection causing pathogen [145] which forms biofilm communities, and thus promotes bacterial persistence [146]. Immunofluorence microscopy analysis after *H. influenzae* infection displayed colocalisation of the NET markers, NE and histones, with the pathogens within the biofilm structure [120]. The co-incubation of live *T. gondii* tachyzoites with harbour seal PMNs resulted in net-like structures with the parasites attached to them [80]. In line with parasite-induced NET formation in other mammalian species [28,60,71] enzymatic activity of NADPH oxidase, NE, and MPO were reported and inhibition of those enzymes resulted in a significant reduction in NETosis [80]. The entrapment of the parasites by seal NETs prevented host cell invasion, thus, displaying a beneficial impact of NETs for the host organism [80]. 

### 3.9. Extracellular Traps in Delphinidae

The release of ETs from dolphins has been demonstrated recently by Villagra-Blanco and colleagues [81]. The apicomplexan parasite *N. caninum,* studied in the context of bovine, canine, and caprine NETs [57,74,127], was used in a study involving neutrophils derived from bottlenose dolphins (Tursiops truncates) [81]. After co-incubation with the parasite for 30 to 60 min, cetacean neutrophils released a fine network of DNA. The parasite-induced NET formation was NOX- and dose-dependent, but not time-dependent. *N. caninum* tachyzoites were found entrapped in a meshwork of DNA filaments or attached to single activated neutrophils [81]. 

### 3.10. Extracellular Traps in Didelphidae

Belonging to the mammalian class, marsupials, however, split from the phylogenetic tree further down, developing a front pouch for carrying their offspring. In a study analysing extracellular traps from granulocytes derived from domesticated dogs, granulocytes from wild opossums *Didelphis (D.) marsupialis* were used as a comparison [77]. Analysis of the cells revealed a high percentage of eosinophils in the granulocyte fraction. Free extracellular DNA was detected after didelphine granulocytes were stimulated by *T. cruzi* [77]. The addition of cholesterol-depleting methyl-β-cyclodextrin (CD) also induced the release of extracellular traps, as has been previously shown in human [31] and bovine neutrophils [126], indicating a conserved role of lipid membrane composition in NET formation. Neutrophil elastase was found within the fibres, after stimulation with both *T. cruzi* and CD. Three-dimensional (3D) imaging further revealed an accumulation of *T. cruzi* within the trap formation [77]. 

## 4. Extracellular Traps of Avian Origin

Chuammitri and colleagues previously suggested, several years ago, that the release of extracellular traps was a conserved host defence mechanism as it was described in various phylogenetically diverse animal species [83]. Heterophils, the avian counterpart to mammalian neutrophil granulocytes [147], were found to release their cellular content in response to PMA and H_2_O_2_ [83]. The capacity of heterophil trap formation (HET) varied in the genetically different chicken lines, with the noncommercially used Fayoumi line displaying the highest HET release in response to H_2_O_2_ as compared with broiler and Leghorn lines [82]. Further genetic analysis revealed that single nucleotide polymorphisms (SNPs) had an effect on the formation of HETs in different chicken lines [148]. A marker on chromosome 5 that had been associated with resistance to Salmonella was found to have an impact on HET production [148]. Additionally, a novel region on chromosome 6 was also significantly involved in HET release [148]. Another study revealed that *S. enterica subsp. enterica* serovars Infantis (SI) and Enteritidis (SE) induced HETs within 15 min of co-incubation in vitro [84]. In addition, Salmonella-derived LPS induced HET formation after 60 min. Both bacterial strains were found entrapped within the NET meshwork, however the entrapped bacteria were not killed by the HETs [84]. 

A recent study reported that the mycotoxin ochratoxin, a strong cytotoxic food contaminant [149] produced by Aspergillus and Penicillium species, triggered the release of chicken HETs [150]. This HET formation was mediated via NADPH oxidases, ERK1/2 and p38 MAPK, and could give insights into hyporeactivity of the immune system to infection due to exposure to ochratoxin [150]. 

## 5. Extracellular Traps in Teleosts

Evolutionary older species, such as fish, also have an advanced and complex immune system, however, the innate immune component plays a more prominent role [151]. NETs were first described in fathead minnow (*Pimephales promelas*) in 2007, by Palic and colleagues [152], and further studies revealed their presence also in zebrafish (*Danio rerio;* [85]), carp (*Cyprinus carpio* [86] and Figure 1), and Turbot (*Scophthalmus maximus;* [87]) in in vitro experiments. However, the function of fish NETs appears to be conserved, at least in teleosts, where neutrophils were shown to rapidly release NETs in a time-dependent manner after just 15 min [153]. Furthermore, these NETs were also capable of entrapping, but not killing the gram-Negative bacteria *A. hydrophila* [86]. As shown in mammals, NETosis can be induced in carp neutrophils with various stimuli, such as PMA, zymosan/ beta-glucan, *P. fluorescens*, *Vibrio harveyi*, *C. albicans*, Poly I:C, and LPS, demonstrating the potential broad scope of action they have on fungi, viruses, and bacteria [88,89,90,153]. Furthermore, as described in humans [154], carp NETs were induced in a ROS independent and dependent manner [88]. In addition to ROS involvement, increased levels of MPO and NO were found in tongue sole NETs, when stimulated with PMA, *P. fluorescens*, or *Vibrio harveyi* [89]. The NETs analysed, in this study, were released from viable neutrophils, suggesting a non-cell death pathway of NETosis in teleost [89]. Further studies by the same group found histones and chymotrypsin-like elastases to be involved in the antimicrobial activity of those teleost ETs [155]. Gratacap and colleagues used a transparent zebrafish swim bladder infection model to analyse NET formation [90]. Their study suggested that ETs play a key role in damaging fungal filaments and limiting the extent of invasion when teleost where infected with *C. albicans* [90]. 

The antimicrobial function of the DNA backbone in piscine NETs has been illustrated with the ability of *A. hydrophila* to degrade the DNA structures via nucleases [86]. Turbot (*Scophthalmus maximus*) NETs have been shown to entrap both *P. fluorescens* and *E. coli*, and additionally kill *E. coli* [87]. Furthermore, the authors showed that degradation of NETs via DNase incubation led to an increase in bacterial load as compared with an intact NET control [89].

## 6. Extracellular Traps in Invertebrates

In addition to vertebrates, recent studies have shown the ability of (aquatic) invertebrates including the blue mussel (*Mytilus edulis*), slug species (*Arion lusitanicus* and *Limax maximus*), snail (*Achatina fulicato*), and shore crab (*Carcinus maenas*) release chromatin-based ETs, termed invertebrate extracellular phagocyte traps (EPTs) [6,156]. Although the species discussed here are derived from different phyla, we decided to group them in this chapter, since studies on invertebrates have remained sparse and mostly limited to Arthropoda (crabs, shrimps) and Mollusca (mussel, snails, and slugs) [6,91,92,93,94,156].

Extracellular chromatin originating from the nucleus and coated in histones has been described in Pacific white shrimp (*Litopenaeus vannamei*) [91]. The authors visualise ETs, 30 min after stimulation with PMA, LPS, and *E. coli* which were capable of entrapping and potentially killing *E. coli* [91]. An in vitro study showed that hemocytes from the kuruma shrimp (arthropod) release ETs upon challenge with LPS, which are able to entrap bacteria [156]. 

Haemocytes isolated from slugs and snails responded to larval stages of canine and feline metastrongloid lungworms with extracellular DNA structures in vitro and in vivo, ensnaring the parasites [93]. Despite the strong entrapment of the worms, viability seemed unaffected. The released fibres stained positive for H1, H2A/B, H3, H4, and MPO and were detected within 10 min of co-incubation [93]. The authors suggested that ET formation is a general effector mechanism, independent of parasite species, parasite stage, or haemocyte origin [93]. Phylogenetic comparison by different authors confirmed that histones have been shown to be crucial components of mammalian and fish extracellular traps [151,153,157] and are also major antimicrobial effectors against bacteria in oyster gills [94]. A rapid release of extracellular DNA after 1 h was observed, which was shown to ensnare numerous pathogens [94]. 

Interestingly, cells from the sea anemone *Actinia equina* (Cnidaria) also released DNA fibres in response to a chemical stimuli (PMA) [6]. These data indicate that ETs are shared by relatively distant species belonging to different branches of the Bilateria, such as Chordata (mammals, birds, and fish), Arthropoda, and Mollusca (shrimp, oyster, and mussel) and even Cnidaria. Furthermore, in 2016, Zhang et al. identified DNA traps released by the social amoebae *Dictyostelium discoideum* (Amoebozoa) that are also able to kill bacteria [158]. 

## 7. Extracellular Traps in Plants

It is also important to highlight that extracellular DNA traps are required for root tip resistance of plants to fungal infections in a similar way as shown for mammalian NETs. On the basis of the initial work by Wen et al. who described the importance of root tip extracellular DNA in protecting plants against fungal infections [159], Hawes et al. hypothesised that extracellular DNA present on border root cells play an immunological role against bacterial pathogens [160,161]. In 2016, these findings were supported by the results of Tran et al. who showed that extracellular DNases from the plant pathogenic bacterium *Ralstonia solanacearum* degrade these extracellular traps and contribute to virulence [162]. Wen and coworkers showed that root tips of *Zea mays* display DNA-positive strands within 1 to 2 min after immersion in water [163]. The structures disappeared when treated with DNAse I [163]. Pea cells (*Pisum sativum*) released extracellular DNA fibres after 2 h when incubated in water. The DNA staining was visible surrounding the border cells and surface of the peripheral root cap, with no staining inside the border cells. Interestingly, recent findings revealed that soil contaminants can be entrapped within extracellular DNA released by root border cells [164]. The authors proposed that a better understanding of border cell ETs would help develop a non-destructive approach to neutralise environmental threats [164].

## 8. Discussion

As summarised in this review, the conserved nature of ETs throughout multiple kingdoms gives clues to their function and underlying mechanisms involved in their formation and modulation. Recent research in mammals associated ETs with autoimmune disorders, as well as several other detrimental effects, however, only a few studies have been conducted in other animal classes. Furthermore, due to similarities in morphology, biochemical pathways, and functional capabilities, fish neutrophils are also highly likely to be involved in autoimmune reactions [165]. However, further research needs to be conducted in this area to confirm if ETs also need to be considered as potential players in autoimmune situations of different animals. 

Two additional conserved aspects include the composition and the stimuli capable of inducing NETs. Studies that have attempted to quantify ETs via software programming [166,167] defined NETs from human neutrophils as changes in DNA/chromatin staining pattern [168]. ETs have been described at around 153 nm in length as a bead-on-a-string model [169]. Stimuli vary from a range of bacterial, viral, or parasitic pathogens, endogenous molecules to chemical compounds or trauma. Extracellular traps have been mostly characterised as extracellular DNA, associated with histones and granular proteins. Although the amount of associated proteins and the composition varies, depending on the origin of the host cells and the stimulus investigated, it could be suggested that ET is defined as a mixture of DNA and cell-specific proteins. 

Taken together, these publications support the concept of an evolutionary conserved immune mechanism without ruling out the hypothesis of a convergent evolution between phylogenetically distant species [94]. Thus, the question remains if organisms from other kingdoms of life use a similar mechanism as defence strategies against its foes. Several bacteria, especially those that are able to form biofilms, such as *Pseudomonas aeruginosa* [121], use extracellular DNA which is used to form biofilms and mediates resistance against antimicrobial effectors or antibiotics. However, this eDNA derived from bacteria is not able to from fibres that look similar to ETs from eukaryotic cells. It is possible that the granule proteins associated with the nuclear or mitochondrial DNA from eukaryotic cells are fine-tuning the composition for its increased stability, as described for the cationic antimicrobial peptides of the cathelicidin family [170]. 

When comparing the efficiency of different immune cells derived from different species, it remains to be determined, and directly compared, which cell types are releasing ETs more strongly as compared with other cell types. Our own data have shown that cholesterol and sphingomyelin are key factors in modulating NET release of neutrophils [31]. Thus, it is possible that differences in the membrane lipid composition of neutrophils derived from different animal species could explain the cell-specific NET-phenotype. In line with this hypothesis, it was reported that different subpopulations of neutrophils, including low-density neutrophils/LDN, express higher susceptibility towards NET stimuli, for example, in the case of lupus [138] or CD16^high^ CD62L^dim^ neutrophils in regard to cancer [107]. The latter study suggested that this specific subset of cells could be correlated to increased survival of the patients suffering from head and neck squamous cell carcinoma (HNSCC) [107]. 

However, a question remains regarding the importance of this immune mechanism within the whole arsenal available for each species. It is hypothesised that more ancient species, including reptiles, such as gopher tortoises [171] and invertebrates, are more dependent on NETs as a form of defence against invading pathogens as compared with species possessing a more complex and developed immune system.

## 9. Conclusions

Since their discovery in 2004, research in ETs has grown exponentially, with an increasing number of species and cell types able to employ this host defence mechanism, as summarised in this review. As a result of the development of sophisticated state-of-the-art microscopy techniques, ETs have been identified as an ancient weapon which has been used by several kingdoms of life for their defence against a variety of pathogens. Understanding this evolutionary history of ET formation would help us modify this innate defence as a target to modulate autoimmunity, infections, thrombosis, cancer, or other diseases.

## Figures and Tables

**Table 1:** Phylum and family origin of extracellular traps (ETs) in the kingdom of Animalia with examples for ET-inducer. Plants are not included.

Phylum	Family	ET Inducer	Reference
Chordata	Humans	*E. coli*, LPS, IL-8, *S. aureus*, Cyclodextrin, *A. fumigatus*, *B. burgdorferi*, *GPAC*, HIV, Myxoma virus, *T. gondii*, PMA, *Entamoeba histolytica*, *H. influenzae*	[1,22,23,24,25,26,27,28,29,30,31,32]
	Mice, Rats, Chinchilla	*C. albicans* hyphae, *L. monocytogenes*, *S. pneumoniae*, LPS, *T. gondii* tachyzoites, *S. stercoralis* larvae, PMA, PAF, ionomycin, lupus serum components, Influenza virus, PTU, Ischemia/Reperfusion, *M. tuberculosis*	[5,9,33,34,35,36,37,38,39,40,41,42,43,44]
	Cows, Sheep, Goats	*M. haemolytica*, MPEC, *E. faecalis*, *S. aureus*, *S. marcecens*, *H. somni*, *S. dysgalactiae*, *E. bovis*, *E. arloingi* sporozoites, Enrofloxacin, *B. besnoiti*, *C. parvum*, Spermatozoa, *H. contortus*, *N. caninum*, D(-)lactic acid, *M. bovis*, *E. ninakohlyakimovae*, *T. gondii*, *S. uberis*	[45,46,47,48,49,50,51,52,53,54,55,56,57,58,59,60,61,62,63,64]
	Pigs	*S. aureus*, *P. aeruginosa*, *S. suis*, PMA, ionomycin, zymosan	[65,66,67]
	Horses, Donkeys	*E. coli*, *S. equi subsp. zooepidermicus*, *S. capitis*, spermatozoa, PMA, *T. gondii*	[2,68,69,70]
	Cats	*Leishmania amazonensis* promastigotes, PMA, *T. gondii*, *E. coli*	[71,72,73]
	Dogs	PMA, PAF, *N. caninum* tachyzoites, cyclodextrin, *E. coli* LPS, Nickel (III) nitrate hexahydrate, *T. cruzi*, *D. immitis* microfilariae	[3,72,74,75,76,77,78]
	Ferrets	Influenza A virus	[79]
	Seals	*T. gondii* tachyzoites, *B. besnoiti* tachyzoites, zymosan	[80]
	Dolphins	Zymosan, *N. caninum* tachyzoites	[81]
	Opossum	*T. cruzi*, cyclodextrin	[77]
	Chicken	PMA, H_2_O_2_, *S. enterica*	[82,83,84]
	Carp, fathead minnow, zebrafish, turbot	*A. hydrophila*, *P. flourescens*, *V. harveyi*, PMA, zymosan, beta-glucan, poly I:C, LPS, calcium ionophore, *C. albicans*	[85,86,87,88,89,90]
Arthropoda	Shrimps, Crabs	*V. anguillarum*, LPS, PMA, *E. coli*	[6,91,92]
Mollusca	Mussel, slugs, snails, oyster	*V. tasmaniensis*, *B. stationis*, zymosan,	[93,94]
Cnidaria	Sea anemone	PMA	[6]

## List of Abbreviations

ETs, extracellular traps; LPS, lipopolysaccharide; PMA, phorbol myristate acetate; LDN, low-density neutrophils; NE, neutrophil elastase; MPO, myeloperoxidase; PAD4, peptidyl arginine deiminase 4; ROS, reactive oxygen species; citH3, citrullinated histone 3; PMNs, polymorph nuclear cells; SOCE, store-operated calcium entry; Poly I:C, polyinosinic:polycytidylic acid; NO, nitric oxide; PAF, platelet-activating factor; SCGB, secretoglobin; PBMCs, peripheral blood mononuclear cell; fMLP, N-formylmethionine-leucyl-phenylalanine, MPO AVV, MPO-antineutrophil cytoplasmic antibody (ANCA)-associated vasculitis; PTU, propylthiouracil; MPEC, mammary pathogenic Escherichia coli; BUVECs, bovine umbilical vein endothelial cells; MAPK, mitogen-activated protein kinase; OM, otitis media; NETs, neutrophil extracellular traps; HETs, heterophil extracellular traps; FeLV, feline leukaemia virus; IMHA, immune-mediated haemolytic anaemia; CD, cyclodextrin; SNPs; single nucleotide polymorphisms; SI; *S. enterica subsp. enterica* serovars Infantis; SE; *S. enterica subsp. enterica* serovars Enteritidis; HNSCC; head and neck squamous cell carcinoma; G-CSF, granulocyte-colony stimulating factor; MI/R, myocardial ischemia/reperfusion; SLE, systemic lupus erythematosus; GBS, group B streptococci; HIV, human immunodeficiency viruses, ANCA, antineutrophil cytoplasmic antibody; CGD, chronic granulomatous disease, PBMCs, peripheral blood mononuclear cell, ERU, equine recurrent uveitis.

## References

[1] Brinkmann V., Reichard U., Goosmann C., Fauler B., Uhlemann Y., Weiss D.S., Weinrauch Y., Zychlinsky A. (2004). Neutrophil Extracellular Traps Kill Bacteria. Science.

[2] Rebordão M.R., Carneiro C., Alexandre-Pires G., Brito P., Pereira C., Nunes T., Galvão A., Leitão A., Vilela C., Ferreira-Dias G. (2014). Neutrophil extracellular traps formation by bacteria causing endometritis in the mare. J. Reprod. Immunol..

[3] Jeffery U., Kimura K., Gray R., Lueth P., Bellaire B., LeVine D. (2015). Dogs cast NETs too: Canine neutrophil extracellular traps in health and immune-mediated hemolytic anemia. Vet. Immunol. Immunopathol..

[4] Pisanu S., Cubeddu T., Pagnozzi D., Rocca S., Cacciotto C., Alberti A., Marogna G., Uzzau S., Addis M.F. (2015). Neutrophil extracellular traps in sheep mastitis. Vet. Res..

[5] Bonne-Année S., Kerepesi L.A., Hess J.A., Wesolowski J., Paumet F., Lok J.B., Nolan T.J., Abraham D. (2014). Extracellular traps are associated with human and mouse neutrophil and macrophage mediated killing of larval Strongyloides stercoralis. Microbes Infect..

[6] Robb C.T., Dyrynda E.A., Gray R.D., Rossi A.G., Smith V.J. (2014). Invertebrate extracellular phagocyte traps show that chromatin is an ancient defence weapon. Nat. Commun..

[7] De Buhr N., Von Köckritz-Blickwede M. (2016). How Neutrophil Extracellular Traps Become Visible. J. Immunol. Res..

[8] Daniel C., Leppkes M., Muñoz L.E., Schley G., Schett G., Herrmann M. (2019). Extracellular DNA traps in inflammation, injury and healing. Nat. Rev. Nephrol..

[9] Brill A., Fuchs T.A., Savchenko A.S., Thomas G.M., Martinod K., de Meyer S.F., Bhandari A.A., Wagner D.D. (2012). Neutrophil extracellular traps promote deep vein thrombosis in mice. J. Thromb. Haemost..

[10] Merza M., Hartman H., Rahman M., Hwaiz R., Zhang E., Renström E., Luo L., Mörgelin M., Regner S., Thorlacius H. (2015). Neutrophil Extracellular Traps Induce Trypsin Activation, Inflammation, and Tissue Damage in Mice with Severe Acute Pancreatitis. Gastroenterology.

[11] Lood C., Blanco L.P., Purmalek M.M., Carmona-Rivera C., De Ravin S.S., Smith C.K., Malech H.L., Ledbetter J.A., Elkon K.B., Kaplan M.J. (2016). Neutrophil extracellular traps enriched in oxidized mitochondrial DNA are interferogenic and contribute to lupus-like disease. Nat. Med..

[12] Cools-Lartigue J., Spicer J., Najmeh S., Ferri L. (2014). Neutrophil extracellular traps in cancer progression. Cell. Mol. Life Sci..

[13] Euler M., Hoffmann M.H. (2019). The double-edged role of neutrophil extracellular traps in inflammation. Biochem. Soc. Trans..

[14] Fuchs T.A., Abed U., Goosmann C., Hurwitz R., Schulze I., Wahn V., Weinrauch Y., Brinkmann V., Zychlinsky A. (2007). Novel cell death program leads to neutrophil extracellular traps. J. Cell Biol..

[15] Wartha F., Henriques-Normark B. (2008). ETosis: A novel cell death pathway. Sci. Signal..

[16] Pilsczek F.H., Salina D., Poon K.K.H., Fahey C., Yipp B.G., Sibley C.D., Robbins S.M., Green F.H.Y., Surette M.G., Sugai M. (2010). A Novel Mechanism of Rapid Nuclear Neutrophil Extracellular Trap Formation in Response to Staphylococcus aureus. J. Immunol..

[17] Yipp B.G., Petri B., Salina D., Jenne C.N., Scott B.N.V., Zbytnuik L.D., Pittman K., Asaduzzaman M., Wu K., Meijndert H.C. (2012). Infection-induced NETosis is a dynamic process involving neutrophil multitasking in vivo. Nat. Med..

[18] McIlroy D.J., Jarnicki A.G., Au G.G., Lott N., Smith D.W., Hansbro P.M., Balogh Z.J. (2014). Mitochondrial DNA neutrophil extracellular traps are formed after trauma and subsequent surgery. J. Crit. Care.

[19] Yousefi S., Mihalache C., Kozlowski E., Schmid I., Simon H.U. (2009). Viable neutrophils release mitochondrial DNA to form neutrophil extracellular traps. Cell Death Differ..

[20] Boeltz S., Amini P., Anders H.J., Andrade F., Bilyy R., Chatfield S., Cichon I., Clancy D.M., Desai J., Dumych T. (2019). To NET or not to NET: Current opinions and state of the science regarding the formation of neutrophil extracellular traps. Cell Death Differ..

[21] Kato A., Rooney A.P., Furutani Y., Hirose S. (2010). Evolution of trappin genes in mammals. BMC Evol. Biol..

[22] Halverson T.W.R., Wilton M., Poon K.K.H., Petri B., Lewenza S. (2015). DNA Is an Antimicrobial Component of Neutrophil Extracellular Traps. PLoS Pathog..

[23] Neumann A., Björck L., Frick I.-M. (2020). Finegoldia magna, an Anaerobic Gram-Positive Bacterium of the Normal Human Microbiota, Induces Inflammation by Activating Neutrophils. Front. Microbiol..

[24] Röhm M., Grimm M.J., D’Auria A.C., Almyroudis N.G., Segal B.H., Urban C.F. (2014). NADPH oxidase promotes neutrophil extracellular trap formation in pulmonary aspergillosis. Infect. Immun..

[25] Menten-Dedoyart C., Faccinetto C., Golovchenko M., Dupiereux I., Van Lerberghe P.-B., Dubois S., Desmet C., Elmoualij B., Baron F., Rudenko N. (2012). Neutrophil Extracellular Traps Entrap and Kill Borrelia burgdorferi Sensu Stricto Spirochetes and Are Not Affected by Ixodes ricinus Tick Saliva. J. Immunol..

[26] Saitoh T., Komano J., Saitoh Y., Misawa T., Takahama M., Kozaki T., Uehata T., Iwasaki H., Omori H., Yamaoka S. (2012). Neutrophil extracellular traps mediate a host defense response to human immunodeficiency virus-1. Cell Host Microbe.

[27] Jenne C.N., Wong C.H.Y., Zemp F.J., McDonald B., Rahman M.M., Forsyth P.A., McFadden G., Kubes P. (2013). Neutrophils recruited to sites of infection protect from virus challenge by releasing neutrophil extracellular traps. Cell Host Microbe.

[28] Abdallah D.S.A., Lin C., Ball C.J., King M.R., Duhamel G.E., Denkers E.Y. (2012). Toxoplasma gondii triggers release of human and mouse neutrophil extracellular traps. Infect. Immun..

[29] Kenny E.F., Herzig A., Krüger R., Muth A., Mondal S., Thompson P.R., Brinkmann V., von Bernuth H., Zychlinsky A. (2017). Diverse stimuli engage different neutrophil extracellular trap pathways. eLife.

[30] Ventura-Juarez J., Campos-Esparza M.R., Pacheco-Yepez J., López-Blanco J.A., Adabache-Ortíz A., Silva-Briano M., Campos-Rodríguez R. (2016). Entamoeba histolytica induces human neutrophils to form NETs. Parasite Immunol..

[31] Neumann A., Brogden G., Jerjomiceva N., Brodesser S., Naim H.Y., Von Köckritz-Blickwede M. (2014). Lipid alterations in human blood-derived neutrophils lead to formation of neutrophil extracellular traps. Eur. J. Cell Biol..

[32] Juneau R.A., Pang B., Weimer K.W.D., Armbruster C.E., Swords W.E. (2011). Nontypeable haemophilus influenzae initiates formation of neutrophil extracellular traps. Infect. Immun..

[33] Etulain J., Martinod K., Wong S.L., Cifuni S.M., Schattner M., Wagner D.D. (2015). P-selectin promotes neutrophil extracellular trap formation in mice. Blood.

[34] Koji T., Yuhki K., Tadanobu S., Masato O., Shozo I., Yuji T., Yoshinaga O., Yasuhiro I., Toshimitsu A., Keiichi U. (2014). In vivo characterization of neutrophil extracellular traps in various organs of a murine sepsis model. PLoS ONE.

[35] Knight J.S., Zhao W., Luo W., Subramanian V., O’Dell A.A., Yalavarthi S., Hodgin J.B., Eitzman D.T., Thompson P.R., Kaplan M.J. (2013). Peptidylarginine deiminase inhibition is immunomodulatory and vasculoprotective in murine lupus. J. Clin. Investig..

[36] Boettcher M., Meier D., Jiménez-Alcázar M., Eschenburg G., Mietzsch S., Vincent D., Klinke M., Trochimiuk M., Appl B., Tiemann B. (2017). Degradation of Extracellular DNA by DNase1 Significantly Reduces Testicular Damage After Testicular Torsion in Rats. Urology.

[37] Cedervall J., Zhang Y., Huang H., Zhang L., Femel J., Dimberg A., Olsson A.K. (2015). Neutrophil extracellular traps accumulate in peripheral blood vessels and compromise organ function in tumor-bearing animals. Cancer Res..

[38] Gao X., Hao S., Yan H., Ding W., Li K., Li J. (2015). Neutrophil extracellular traps contribute to the intestine damage in endotoxemic rats. J. Surg. Res..

[39] Savchenko A.S., Borissoff J.I., Martinod K., De Meyer S.F., Gallant M., Erpenbeck L., Brill A., Wang Y., Wagner D.D. (2014). VWF-mediated leukocyte recruitment with chromatin decondensation by PAD4 increases myocardial ischemia/reperfusion injury in mice. Blood.

[40] Ge L., Zhou X., Ji W.J., Lu R.Y., Zhang Y., Zhang Y.D., Ma Y.Q., Zhao J.H., Li Y.M. (2015). Neutrophil extracellular traps in ischemia-reperfusion injury-induced myocardial no-reflow: Therapeutic potential of DNase-based reperfusion strategy. Am. J. Physiol.-Hear. Circ. Physiol..

[41] Nakazawa D., Tomaru U., Suzuki A., Masuda S., Hasegawa R., Kobayashi T., Nishio S., Kasahara M., Ishizu A. (2012). Abnormal conformation and impaired degradation of propylthiouracil-induced neutrophil extracellular traps: Implications of disordered neutrophil extracellular traps in a rat model of myeloperoxidase antineutrophil cytoplasmic antibody-associated vasculiti. Arthritis Rheum..

[42] Ermert D., Urban C.F., Laube B., Goosmann C., Zychlinsky A., Brinkmann V. (2009). Mouse neutrophil extracellular traps in microbial infections. J. Innate Immun..

[43] Beiter K., Wartha F., Albiger B., Normark S., Zychlinsky A., Henriques-Normark B. (2006). An endonuclease allows Streptococcus pneumoniae to escape from neutrophil extracellular traps. Curr. Biol..

[44] Hopke A., Nicke N., Hidu E.E., Degani G., Popolo L., Wheeler R.T. (2016). Neutrophil Attack Triggers Extracellular Trap-Dependent Candida Cell Wall Remodeling and Altered Immune Recognition. PLoS Pathog..

[45] Swain D.K., Kushwah M.S., Kaur M., Patbandha T.K., Mohanty A.K., Dang A.K. (2014). Formation of NET, phagocytic activity, surface architecture, apoptosis and expression of toll like receptors 2 and 4 (TLR2 and TLR4) in neutrophils of mastitic cows. Vet. Res. Commun..

[46] Gondaira S., Higuchi H., Nishi K., Iwano H., Nagahata H. (2017). Mycoplasma bovis escapes bovine neutrophil extracellular traps. Vet. Microbiol..

[47] Caro T.M., Hermosilla C., Silva L.M.R., Cortes H., Taubert A. (2014). Neutrophil extracellular traps as innate immune reaction against the emerging apicomplexan parasite besnoitia besnoiti. PLoS ONE.

[48] Aulik N.A., Hellenbrand K.M., Klos H., Czuprynski C.J. (2010). Mannheimia haemolytica and its leukotoxin cause neutrophil extracellular trap formation by bovine neutrophils. Infect. Immun..

[49] Muñoz-Caro T., Machado Ribeiro da Silva L., Rentería-Solis Z., Taubert A., Hermosilla C. (2016). Neutrophil extracellular traps in the intestinal mucosa of Eimeria-infected animals. Asian Pac. J. Trop. Biomed..

[50] Grinberg N., Elazar S., Rosenshine I., Shpigel N.Y. (2008). β-hydroxybutyrate abrogates formation of bovine neutrophil extracellular traps and bactericidal activity against mammary pathogenic Escherichia coli. Infect. Immun..

[51] Hellenbrand K.M., Forsythe K.M., Rivera-Rivas J.J., Czuprynski C.J., Aulik N.A. (2013). Histophilus somni causes extracellular trap formation by bovine neutrophils and macrophages. Microb. Pathog..

[52] Lippolis J.D., Reinhardt T.A., Goff J.P., Horst R.L. (2006). Neutrophil extracellular trap formation by bovine neutrophils is not inhibited by milk. Vet. Immunol. Immunopathol..

[53] Revelo X.S., Waldron M.R. (2012). In vitro effects of Escherichia coli lipopolysaccharide on the function and gene expression of neutrophils isolated from the blood of dairy cows. J. Dairy Sci..

[54] Behrendt J.H., Ruiz A., Zahner H., Taubert A., Hermosilla C. (2010). Neutrophil extracellular trap formation as innate immune reactions against the apicomplexan parasite Eimeria bovis. Vet. Immunol. Immunopathol..

[55] Muñoz-Caro T., Lendner M., Daugschies A., Hermosilla C., Taubert A. (2015). NADPH oxidase, MPO, NE, ERK1/2, p38 MAPK and Ca2+ influx are essential for Cryptosporidium parvum-induced NET formation. Dev. Comp. Immunol..

[56] Jerjomiceva N., Seri H., Völlger L., Wang Y., Zeitouni N., Naim H.Y., Von Köckritz-Blickwede M. (2014). Enrofloxacin enhances the formation of neutrophil extracellular traps in bovine granulocytes. J. Innate Immun..

[57] Villagra-Blanco R., Silva L.M.R., Muñoz-Caro T., Yang Z., Li J., Gärtner U., Taubert A., Zhang X., Hermosilla C. (2017). Bovine polymorphonuclear neutrophils cast neutrophil extracellular traps against the abortive parasite Neospora caninum. Front. Immunol..

[58] Muñoz-Caro T., Huertas S.J.M., Conejeros I., Alarcón P., Hidalgo M.A., Burgos R.A., Hermosilla C., Taubert A. (2015). Eimeria bovis-triggered neutrophil extracellular trap formation is cd11b-, ERK 1/2-, p38 MAP kinase- and soce-dependent. Vet. Res..

[59] Muñoz-Caro T., Rubio R M.C., Silva L.M.R., Magdowski G., Gärtner U., McNeilly T.N., Taubert A., Hermosilla C. (2015). Leucocyte-derived extracellular trap formation significantly contributes to Haemonchus contortus larval entrapment. Parasites Vectors.

[60] Yildiz K., Gokpinar S., Gazyagci A.N., Babur C., Sursal N., Azkur A.K. (2017). Role of NETs in the difference in host susceptibility to Toxoplasma gondii between sheep and cattle. Vet. Immunol. Immunopathol..

[61] Alarcón P., Manosalva C., Conejeros I., Carretta M.D., Muñoz-Caro T., Silva L.M.R., Taubert A., Hermosilla C., Hidalgo M.A., Burgos R.A. (2017). D(−) lactic acid-induced adhesion of bovine neutrophils onto endothelial cells is dependent on neutrophils extracellular traps formation and CD11b expression. Front. Immunol..

[62] Silva L.M.R., Muñoz Caro T., Gerstberger R., Vila-Viçosa M.J.M., Cortes H.C.E., Hermosilla C., Taubert A. (2014). The apicomplexan parasite Eimeria arloingi induces caprine neutrophil extracellular traps. Parasitol. Res..

[63] Pérez D., Muñoz M.C., Molina J.M., Muñoz-Caro T., Silva L.M.R., Taubert A., Hermosilla C., Ruiz A. (2016). Eimeria ninakohlyakimovae induces NADPH oxidase-dependent monocyte extracellular trap formation and upregulates IL-12 and TNF-α, IL-6 and CCL2 gene transcription. Vet. Parasitol..

[64] Muñoz-Caro T., Silva L.M.R., Ritter C., Taubert A., Hermosilla C. (2014). Besnoitia besnoiti tachyzoites induce monocyte extracellular trap formation. Parasitol. Res..

[65] Bréa D., Meurens F., Dubois A.V., Gaillard J., Chevaleyre C., Jourdan M.L., Winter N., Arbeille B., Si-Tahar M., Gauthier F. (2012). The pig as a model for investigating the role of neutrophil serine proteases in human inflammatory lung diseases. Biochem. J..

[66] De Buhr N., Neumann A., Jerjomiceva N., von Köckritz-Blickwede M., Baums C.G. (2014). Streptococcus suis DNase SsnA contributes to degradation of neutrophil extracellular traps (NETs) and evasion of NET-mediated antimicrobial activity. Microbiology.

[67] Loving C.L., Kehrli M.E., Brockmeier S.L., Bayles D.O., Michael D.D., Schlink S.N., Lager K.M. (2013). Porcine granulocyte-colony stimulating factor (G-CSF) delivered via replication-defective adenovirus induces a sustained increase in circulating peripheral blood neutrophils. Biologicals.

[68] Alghamdi A.S., Foster D.N. (2005). Seminal DNase Frees Spermatozoa Entangled in Neutrophil Extracellular Traps. Biol. Reprod..

[69] Herteman N., Vargas A., Lavoie J.P. (2017). Characterization of Circulating Low-Density Neutrophils Intrinsic Properties in Healthy and Asthmatic Horses. Sci. Rep..

[70] Yildiz K., Gokpinar S., Sursal N., Babur C., Ozen D., Azkur A.K. (2019). Extracellular Trap Formation by Donkey Polymorphonuclear Neutrophils against Toxoplasma gondii. J. Equine Vet. Sci..

[71] Lacerda L.C., dos Santos J.L., Wardini A.B., da Silva A.N., Santos A.G., Silva Freire H.P., dos Anjos D.O., Romano C.C., Mendes É.A., Munhoz A.D. (2019). Toxoplasma gondii induces extracellular traps release in cat neutrophils. Exp. Parasitol..

[72] Rebordão M.R., Alexandre-Pires G., Carreira M., Adriano L., Carneiro C., Nunes T., Mateus L., Ferreira-Dias G. (2017). Bacteria causing pyometra in bitch and queen induce neutrophil extracellular traps. Vet. Immunol. Immunopathol..

[73] Wardini A.B., Guimarães-Costa A.B., Nascimento M.T.C., Nadaes N.R., Danelli M.G.M., Mazur C., Benjamin C.F., Saraiva E.M., Pinto-da-Silva L.H. (2010). Characterization of neutrophil extracellular traps in cats naturally infected with feline leukemia virus. J. Gen. Virol..

[74] Wei Z., Hermosilla C., Taubert A., He X., Wang X., Gong P., Li J., Yang Z., Zhang X. (2016). Canine neutrophil extracellular traps release induced by the apicomplexan parasite Neospora caninum in vitro. Front. Immunol..

[75] Jeffery U., LeVine D.N. (2018). Canine Neutrophil Extracellular Traps Enhance Clot Formation and Delay Lysis. Vet. Pathol..

[76] Li R.H.L., Ng G., Tablin F. (2017). Lipopolysaccharide-induced neutrophil extracellular trap formation in canine neutrophils is dependent on histone H3 citrullination by peptidylarginine deiminase. Vet. Immunol. Immunopathol..

[77] De Buhr N., Bonilla M.C., Jimenez-Soto M., von Köckritz-Blickwede M., Dolz G. (2018). Extracellular trap formation in response to Trypanosoma cruzi infection in granulocytes isolated from dogs and common opossums, natural reservoir hosts. Front. Microbiol..

[78] Muñoz-Caro T., Conejeros I., Zhou E., Pikhovych A., Gärtner U., Hermosilla C., Kulke D., Taubert A. (2018). Dirofilaria immitis microfilariae and third-stage larvae induce canine NETosis resulting in different types of neutrophil extracellular traps. Front. Immunol..

[79] Camp J.V., Bagci U., Chu Y.-K., Squier B., Fraig M., Uriarte S.M., Guo H., Mollura D.J., Jonsson C.B. (2015). Lower Respiratory Tract Infection of the Ferret by 2009 H1N1 Pandemic Influenza A Virus Triggers Biphasic, Systemic, and Local Recruitment of Neutrophils. J. Virol..

[80] Reichel M., Muñoz-Caro T., Sanchez Contreras G., Rubio García A., Magdowski G., Gärtner U., Taubert A., Hermosilla C. (2015). Harbour seal (Phoca vitulina) PMN and monocytes release extracellular traps to capture the apicomplexan parasite Toxoplasma gondii. Dev. Comp. Immunol..

[81] Villagra-Blanco R., Silva L.M.R., Aguilella-Segura A., Arcenillas-Hernández I., Martínez-Carrasco C., Seipp A., Gärtner U., Ruiz de Ybañez R., Taubert A., Hermosilla C. (2017). Bottlenose dolphins (Tursiops truncatus) do also cast neutrophil extracellular traps against the apicomplexan parasite Neospora caninum. Int. J. Parasitol. Parasites Wildl..

[82] Chuammitri P., Redmond S.B., Kimura K., Andreasen C.B., Lamont S.J., Palić D. (2011). Heterophil functional responses to dietary immunomodulators vary in genetically distinct chicken lines. Vet. Immunol. Immunopathol..

[83] Chuammitri P., Ostojić J., Andreasen C.B., Redmond S.B., Lamont S.J., Palić D. (2009). Chicken heterophil extracellular traps (HETs): Novel defense mechanism of chicken heterophils. Vet. Immunol. Immunopathol..

[84] Pieper J., Locke M., Ruzaike G., Voigt S., Methner U., Berndt A. (2017). In vitro and in vivo generation of heterophil extracellular traps after Salmonella exposure. Vet. Immunol. Immunopathol..

[85] Palić D., Andreasen C.B., Ostojić J., Tell R.M., Roth J.A. (2007). Zebrafish (Danio rerio) whole kidney assays to measure neutrophil extracellular trap release and degranulation of primary granules. J. Immunol. Methods.

[86] Brogden G., von Köckritz-Blickwede M., Adamek M., Reuner F., Jung-Schroers V., Naim H.Y., Steinhagen D. (2012). β-Glucan protects neutrophil extracellular traps against degradation by Aeromonas hydrophila in carp (Cyprinus carpio). Fish Shellfish Immunol..

[87] Chi H., Sun L. (2016). Neutrophils of Scophthalmus maximus produce extracellular traps that capture bacteria and inhibit bacterial infection. Dev. Comp. Immunol..

[88] Pijanowski L., Golbach L., Kolaczkowska E., Scheer M., Verburg-van Kemenade B.M.L., Chadzinska M. (2013). Carp neutrophilic granulocytes form extracellular traps via ROS-dependent and independent pathways. Fish Shellfish Immunol..

[89] Zhao M.L., Chi H., Sun L. (2017). Neutrophil extracellular traps of Cynoglossus semilaevis: Production characteristics and antibacterial effect. Front. Immunol..

[90] Gratacap R.L., Scherer A.K., Seman B.G., Wheeler R.T. (2017). Control of mucosal candidiasis in the zebrafish swim bladder depends on neutrophils that block filament invasion and drive extracellular-trap production. Infect. Immun..

[91] Ng T.H., Chang S.H., Wu M.H., Wang H.C. (2013). Shrimp hemocytes release extracellular traps that kill bacteria. Dev. Comp. Immunol..

[92] Ng T.H., Wu M.H., Chang S.H., Aoki T., Wang H.C. (2015). The DNA fibers of shrimp hemocyte extracellular traps are essential for the clearance of Escherichia coli. Dev. Comp. Immunol..

[93] Lange M.K., Penagos-Tabares F., Muñoz-Caro T., Gärtner U., Mejer H., Schaper R., Hermosilla C., Taubert A. (2017). Gastropod-derived haemocyte extracellular traps entrap metastrongyloid larval stages of Angiostrongylus vasorum, Aelurostrongylus abstrusus and Troglostrongylus brevior. Parasites Vectors.

[94] Poirier A.C., Schmitt P., Rosa R.D., Vanhove A.S., Kieffer-Jaquinod S., Rubio T.P., Charrière G.M., Destoumieux-Garzón D. (2014). Antimicrobial histones and DNA traps in invertebrate immunity: Evidences in Crassostrea gigas. J. Biol. Chem..

[95] Branzk N., Lubojemska A., Hardison S.E., Wang Q., Gutierrez M.G., Brown G.D., Papayannopoulos V. (2014). Neutrophils sense microbe size and selectively release neutrophil extracellular traps in response to large pathogens. Nat. Immunol..

[96] Hashiba M., Huq A., Tomino A., Hirakawa A., Hattori T., Miyabe H., Tsuda M., Takeyama N. (2015). Neutrophil extracellular traps in patients with sepsis. J. Surg. Res..

[97] Park S.Y., Shrestha S., Youn Y.J., Kim J.K., Kim S.Y., Kim H.J., Park S.H., Ahn W.G., Kim S., Lee M.G. (2017). Autophagy primes neutrophils for neutrophil extracellular trap formation during sepsis. Am. J. Respir. Crit. Care Med..

[98] Lapponi M.J., Carestia A., Landoni V.I., Rivadeneyra L., Etulain J., Negrotto S., Pozner R.G., Schattner M. (2013). Regulation of neutrophil extracellular trap formation by anti-inflammatory drugs. J. Pharmacol. Exp. Ther..

[99] Schauer C., Janko C., Munoz L.E., Zhao Y., Kienhöfer D., Frey B., Lell M., Manger B., Rech J., Naschberger E. (2014). Aggregated neutrophil extracellular traps limit inflammation by degrading cytokines and chemokines. Nat. Med..

[100] Kaplan M.J., Radic M. (2012). Neutrophil Extracellular Traps: Double-Edged Swords of Innate Immunity. J. Immunol..

[101] Czaikoski P.G., Mota J.M.S.C., Nascimento D.C., Sônego F., Castanheira F.V.E.S., Melo P.H., Scortegagna G.T., Silva R.L., Barroso-Sousa R., Souto F.O. (2016). Neutrophil extracellular traps induce organ damage during experimental and clinical sepsis. PLoS ONE.

[102] Zabieglo K., Majewski P., Majchrzak-Gorecka M., Wlodarczyk A., Grygier B., Zegar A., Kapinska-Mrowiecka M., Naskalska A., Pyrc K., Dubin A. (2015). The inhibitory effect of secretory leukocyte protease inhibitor (SLPI) on formation of neutrophil extracellular traps. J. Leukoc. Biol..

[103] Kolaczkowska E., Jenne C.N., Surewaard B.G.J., Thanabalasuriar A., Lee W.Y., Sanz M.J., Mowen K., Opdenakker G., Kubes P. (2015). Molecular mechanisms of NET formation and degradation revealed by intravital imaging in the liver vasculature. Nat. Commun..

[104] Schreiber A., Rousselle A., Becker J.U., Von Mässenhausen A., Linkermann A., Kettritz R. (2017). Necroptosis controls NET generation and mediates complement activation, endothelial damage, and autoimmune vasculitis. Proc. Natl. Acad. Sci. USA.

[105] Donis-Maturano L., Sánchez-Torres L.E., Cerbulo-Vázquez A., Chacón-Salinas R., García-Romo G.S., Orozco-Uribe M.C., Yam-Puc J.C., González-Jiménez M.A., Paredes-Vivas Y.L., Calderón-Amador J. (2015). Prolonged exposure to neutrophil extracellular traps can induce mitochondrial damage in macrophages and dendritic cells. Springerplus.

[106] Ebrahimi F., Giaglis S., Hahn S., Blum C.A., Baumgartner C., Kutz A., Van Breda S.V., Mueller B., Schuetz P., Christ-Crain M. (2018). Markers of neutrophil extracellular traps predict adverse outcome in communityacquired pneumonia: Secondary analysis of a randomised controlled trial. Eur. Respir. J..

[107] Millrud C.R., Kågedal Å., Kumlien Georén S., Winqvist O., Uddman R., Razavi R., Munck-Wikland E., Cardell L.O. (2017). NET-producing CD16high CD62Ldim neutrophils migrate to tumor sites and predict improved survival in patients with HNSCC. Int. J. Cancer.

[108] Boone B.A., Murthy P., Miller-Ocuin J., Doerfler W.R., Ellis J.T., Liang X., Ross M.A., Wallace C.T., Sperry J.L., Lotze M.T. (2018). Chloroquine reduces hypercoagulability in pancreatic cancer through inhibition of neutrophil extracellular traps. BMC Cancer.

[109] Perlman R.L. (2016). Mouse Models of Human Disease: An Evolutionary Perspective. Evol. Med. Public Health.

[110] Akong-Moore K., Chow O.A., von Köckritz-Blickwede M., Nizet V. (2012). Influences of chloride and hypochlorite on neutrophil extracellular trap formation. PLoS ONE.

[111] Neumann A., Papareddy P., Herwald H. (2018). Immunoregulation of Neutrophil Extracellular Trap Formation by Endothelial-Derived p33 (gC1q Receptor). J. Innate Immun..

[112] Clark S.R., Ma A.C., Tavener S.A., McDonald B., Goodarzi Z., Kelly M.M., Patel K.D., Chakrabarti S., McAvoy E., Sinclair G.D. (2007). Platelet TLR4 activates neutrophil extracellular traps to ensnare bacteria in septic blood. Nat. Med..

[113] Hakkim A., Fürnrohr B.G., Amann K., Laube B., Abed U.A., Brinkmann V., Herrmann M., Voll R.E., Zychlinsky A. (2010). Impairment of neutrophil extracellular trap degradation is associated with lupus nephritis. Proc. Natl. Acad. Sci. USA.

[114] Lande R., Ganguly D., Facchinetti V., Frasca L., Conrad C., Gregorio J., Meller S., Chamilos G., Sebasigari R., Riccieri V. (2011). Neutrophils activate plasmacytoid dendritic cells by releasing self-DNA-peptide complexes in systemic lupus erythematosus. Sci. Transl. Med..

[115] Garcia-Romo G.S., Caielli S., Vega B., Connolly J., Allantaz F., Xu Z., Punaro M., Baisch J., Guiducci C., Coffman R.L. (2011). Netting neutrophils are major inducers of type I IFN production in pediatric systemic lupus erythematosus. Sci. Transl. Med..

[116] Watanabe H., Watanabe K.S., Liu K., Hiramatsu S., Zeggar S., Katsuyama E., Tatebe N., Akahoshi A., Takenaka F., Hanada T. (2017). Anti-high Mobility Group Box 1 Antibody Ameliorates Albuminuria in MRL/lpr Lupus-Prone Mice. Mol. Ther. Methods Clin. Dev..

[117] Wada N., Mukai M., Kohno M., Notoya A., Ito T., Yoshioka N. (2002). Prevalence of serum anti-myeloperoxidase antineutrophil cytoplasmic antibodies (MPO-ANCA) in patients with Graves’ disease treated with propylthiouracil and thiamazole. Endocr. J..

[118] Wang Y., Wang W., Wang N., Tall A.R., Tabas I. (2017). Mitochondrial Oxidative Stress Promotes Atherosclerosis and Neutrophil Extracellular Traps in Aged Mice. Arterioscler. Thromb. Vasc. Biol..

[119] Reid S.D., Hong W., Dew K.E., Winn D.R., Pang B., Watt J., Glover D.T., Hollingshead S.K., Swords W.E. (2009). Streptococcus pneumoniae Forms Surface-Attached Communities in the Middle Ear of Experimentally Infected Chinchillas. J. Infect. Dis..

[120] Hong W., Juneau R.A., Pang B., Swords W.E. (2009). Survival of bacterial biofilms within neutrophil extracellular traps promotes nontypeable haemophilus influenzae persistence in the chinchilla model for otitis media. J. Innate Immun..

[121] Wilton M., Charron-Mazenod L., Moore R., Lewenza S. (2016). Extracellular DNA acidifies biofilms and induces aminoglycoside resistance in *Pseudomonas aeruginosa*. Antimicrob. Agents Chemother..

[122] Filio-Rodríguez G., Estrada-García I., Arce-Paredes P., Moreno-Altamirano M.M., Islas-Trujillo S., Ponce-Regalado M.D., Rojas-Espinosa O. (2017). In vivo induction of neutrophil extracellular traps by Mycobacterium tuberculosis in a Guinea pig model. Innate Immun..

[123] FAO (2015). FAO Statistical Pocketbook 2015: World Food and Agriculture.

[124] Alghamdi A.S., Lovaas B.J., Bird S.L., Lamb G.C., Rendahl A.K., Taube P.C., Foster D.N. (2009). Species-specific interaction of seminal plasma on sperm-neutrophil binding. Anim. Reprod. Sci..

[125] Neeli I., Dwivedi N., Khan S., Radic M. (2009). Regulation of extracellular chromatin release from neutrophils. J. Innate Immun..

[126] Baien S.H., Langer M.N., Heppelmann M., von Köckritz-Blickwede M., de Buhr N. (2018). Comparison between K3EDTA and lithium heparin as anticoagulant to isolate bovine granulocytes from blood. Front. Immunol..

[127] Villagra-Blanco R., Silva L.M.R., Gärtner U., Wagner H., Failing K., Wehrend A., Taubert A., Hermosilla C. (2017). Molecular analyses on Neospora caninum-triggered NETosis in the caprine system. Dev. Comp. Immunol..

[128] Wang Y., Li M., Stadler S., Correll S., Li P., Wang D., Hayama R., Leonelli L., Han H., Grigoryev S.A. (2009). Histone hypercitrullination mediates chromatin decondensation and neutrophil extracellular trap formation. J. Cell Biol..

[129] VanderWaal K., Deen J. (2018). Global trends in infectious diseases of swine. Proc. Natl. Acad. Sci. USA.

[130] De Buhr N., Bonilla M.C., Pfeiffer J., Akhdar S., Schwennen C., Kahl B.C., Waldmann K.H., Valentin-Weigand P., Hennig-Pauka I., von Köckritz-Blickwede M. (2019). Degraded neutrophil extracellular traps promote the growth of Actinobacillus pleuropneumoniae. Cell Death Dis..

[131] Sassu E.L., Bossé J.T., Tobias T.J., Gottschalk M., Langford P.R., Hennig-Pauka I. (2018). Update on Actinobacillus pleuropneumoniae—Knowledge, gaps and challenges. Transbound. Emerg. Dis..

[132] Côté O., Clark M.E., Viel L., Labbé G., Seah S.Y.K., Khan M.A., Douda D.N., Palaniyar N., Bienzle D. (2014). Secretoglobin 1A1 and 1A1A differentially regulate neutrophil reactive oxygen species production, phagocytosis and extracellular trap formation. PLoS ONE.

[133] Johansson S., Andersson K., Wennergren G., Wennerås C., Rudin A. (2009). CC16 inhibits the migration of eosinophils towards the formyl peptide fMLF but not towards PGD2. Inflammation.

[134] Katavolos P., Ackerley C.A., Clark M.E., Bienzle D. (2011). Clara cell secretory protein increases phagocytic and decreases oxidative activity of neutrophils. Vet. Immunol. Immunopathol..

[135] Ma F., Guo X., Fan H. (2017). Extracellular nucleases of Streptococcus equi subsp. zooepidemicus degrade neutrophil extracellular traps and impair macrophage activity of the host. Appl. Environ. Microbiol..

[136] Derré-Bobillot A., Cortes-Perez N.G., Yamamoto Y., Kharrat P., Couvé E., Da Cunha V., Decker P., Boissier M.C., Escartin F., Cesselin B. (2013). Nuclease A (Gbs0661), an extracellular nuclease of Streptococcus agalactiae, attacks the neutrophil extracellular traps and is needed for full virulence. Mol. Microbiol..

[137] Alghamdi A., Troedsson M.H.T., Laschkewitsch T., Xue J.L. (2001). Uterine secretion from mares with post-breeding endometritis alters sperm motion characteristics in vitro. Theriogenology.

[138] Villanueva E., Yalavarthi S., Berthier C.C., Hodgin J.B., Khandpur R., Lin A.M., Rubin C.J., Zhao W., Olsen S.H., Klinker M. (2011). Netting Neutrophils Induce Endothelial Damage, Infiltrate Tissues, and Expose Immunostimulatory Molecules in Systemic Lupus Erythematosus. J. Immunol..

[139] Fingerhut L., Ohnesorge B., von Borstel M., Schumski A., Strutzberg-Minder K., Mörgelin M., Deeg C.A., Haagsman H.P., Beineke A., von Köckritz-Blickwede M. (2019). Neutrophil Extracellular Traps in the Pathogenesis of Equine Recurrent Uveitis (ERU). Cells.

[140] Vargas A., Boivin R., Cano P., Murcia Y., Bazin I., Lavoie J.P. (2017). Neutrophil extracellular traps are downregulated by glucocorticosteroids in lungs in an equine model of asthma. Respir. Res..

[141] Carmona-Rivera C., Kaplan M.J. (2016). Induction and quantification of NETosis. Curr. Protoc. Immunol..

[142] Smith S.A., Lawson C.M., McMichael M.A., Jung K., O’Brien M., Achiel R. (2017). Evaluation of assays for quantification of DNA in canine plasma as an indirect marker of NETosis. Vet. Clin. Pathol..

[143] Wei Z., Zhang X., Wang Y., Wang J., Fu Y., Yang Z. (2018). Nickel (II) nitrate hexahydrate triggered canine neutrophil extracellular traps release in vitro. Chemosphere.

[144] Belser J.A., Barclay W., Barr I., Fouchier R.A.M., Matsuyama R., Nishiura H., Peiris M., Russell C.J., Subbarao K., Zhu H. (2018). Ferrets as models for influenza virus transmission studies and pandemic risk assessments. Emerg. Infect. Dis..

[145] Klein J.O. (2000). The burden of otitis media. Vaccine.

[146] Bakaletz L.O. (2007). Bacterial Biofilms in Otitis Media. Pediatr. Infect. Dis. J..

[147] Harmon B.G. (1998). Avian Heterophils in Inflammation and Disease Resistance. Poult. Sci..

[148] Redmond S.B., Chuammitri P., Andreasen C.B., Palić D., Lamont S.J. (2011). Genetic control of chicken heterophil function in advanced intercross lines: Associations with novel and with known Salmonella resistance loci and a likely mechanism for cell death in extracellular trap production. Immunogenetics.

[149] Al-Anati L., Petzinger E. (2006). Immunotoxic activity of ochratoxin A. J. Vet. Pharmacol. Ther..

[150] Han Z., Zhang Y., Wang C., Liu X., Jiang A., Liu Z., Wang J., Yang Z., Wei Z. (2019). Ochratoxin A-Triggered Chicken Heterophil Extracellular Traps Release through Reactive Oxygen Species Production Dependent on Activation of NADPH Oxidase, ERK, and p38 MAPK Signaling Pathways. J. Agric. Food Chem..

[151] Uribe C., Folch H., Enriquez R., Moran G. (2011). Innate and adaptive immunity in teleost fish: A review. Vet. Med. (Praha).

[152] Palić D., Ostojić J., Andreasen C.B., Roth J.A. (2007). Fish cast NETs: Neutrophil extracellular traps are released from fish neutrophils. Dev. Comp. Immunol..

[153] Brogden G., Krimmling T., Adamek M., Naim H.Y., Steinhagen D., Von Köckritz-Blickwede M. (2014). The effect of β-glucan on formation and functionality of neutrophil extracellular traps in carp (Cyprinus carpio L.). Dev. Comp. Immunol..

[154] Yost C.C., Cody M.J., Harris E.S., Thornton N.L., McInturff A.M., Martinez M.L., Chandler N.B., Rodesch C.K., Albertine K.H., Petti C.A. (2009). Impaired neutrophil extracellular trap (NET) formation: A novel innate immune deficiency of human neonates. Blood.

[155] Wen L.-L., Zhao M.-L., Chi H., Sun L. (2018). Histones and chymotrypsin-like elastases play significant roles in the antimicrobial activity of tongue sole neutrophil extracellular traps. Fish Shellfish Immunol..

[156] Koiwai K., Alenton R.R.R., Kondo H., Hirono I. (2016). Extracellular trap formation in kuruma shrimp (Marsupenaeus japonicus) hemocytes is coupled with c-type lysozyme. Fish Shellfish Immunol..

[157] Brinkmann V., Zychlinsky A. (2012). Neutrophil extracellular traps: Is immunity the second function of chromatin?. J. Cell Biol..

[158] Zhang X., Zhuchenko O., Kuspa A., Soldati T. (2016). Social amoebae trap and kill bacteria by casting DNA nets. Nat. Commun..

[159] Wen F., White G.J., Vanetten H.D., Xiong Z., Hawes M.C. (2009). Extracellular DNA is required for root tip resistance to fungal infection. Plant Physiol..

[160] Hawes M.C., Curlango-Rivera G., Wen F., White G.J., VanEtten H.D., Xiong Z. (2011). Extracellular DNA: The tip of root defenses?. Plant Sci..

[161] Hawes M., Allen C., Turgeon B.G., Curlango-Rivera G., Minh Tran T., Huskey D.A., Xiong Z. (2016). Root Border Cells and Their Role in Plant Defense. Annu. Rev. Phytopathol..

[162] Tran T.M., MacIntyre A., Hawes M., Allen C. (2016). Escaping Underground Nets: Extracellular DNases Degrade Plant Extracellular Traps and Contribute to Virulence of the Plant Pathogenic Bacterium Ralstonia solanacearum. PLoS Pathog..

[163] Wen F., Curlango-Rivera G., Huskey D.A., Xiong Z., Hawes M.C. (2017). Visualization of extracellular DNA released during border cell separation from the root cap. Am. J. Bot..

[164] Hawes M.C., McLain J., Ramirez-Andreotta M., Curlango-Rivera G., Flores-Lara Y., Brigham L.A. (2016). Extracellular trapping of soil contaminants by root border cells: New insights into plant defense. Agronomy.

[165] Leiding J.W. (2017). Neutrophil evolution and their diseases in humans. Front. Immunol..

[166] Coelho L.P., Pato C., Friães A., Neumann A., Von Köckritz-Blickwede M., Ramirez M., Carriço J.A. (2015). Automatic determination of NET (neutrophil extracellular traps) coverage in fluorescent microscopy images. Bioinformatics.

[167] Mohanty T., Sørensen O.E., Nordenfelt P. (2018). NETQUANT: Automated quantification of neutrophil extracellular traps. Front. Immunol..

[168] Brinkmann V., Goosmann C., Kühn L.I., Zychlinsky A. (2012). Automatic quantification of in vitro NET formation. Front. Immunol..

[169] Pires R.H., Felix S.B., Delcea M. (2016). The architecture of neutrophil extracellular traps investigated by atomic force microscopy. Nanoscale.

[170] Neumann A., Völlger L., Berends E.T.M., Molhoek E.M., Stapels D.A.C., Midon M., Friães A., Pingoud A., Rooijakkers S.H.M., Gallo R.L. (2014). Novel role of the antimicrobial peptide LL-37 in the protection of neutrophil extracellular Traps against degradation by bacterial nucleases. J. Innate Immun..

[171] Stacy N.I., Fredholm D.V., Rodriguez C., Castro L., Harvey J.W. (2017). Whip-like heterophil projections in consecutive blood films from an injured gopher tortoise (Gopherus polyphemus) with systemic inflammation. Vet. Q..

